# Beneficial immune-modulatory effects of the N-163 strain of *Aureobasidium pullulans*-produced 1,3-1,6 Beta glucans in Duchenne muscular dystrophy: Results of an open-label, prospective, exploratory case-control clinical study

**DOI:** 10.1016/j.ibneur.2023.06.007

**Published:** 2023-07-04

**Authors:** Kadalraja Raghavan, Vidyasagar Devaprasad Dedeepiya, Subramaniam Srinivasan, Subramanian Pushkala, Sudhakar S. Bharatidasan, Nobunao Ikewaki, Masaru Iwasaki, Rajappa Senthilkumar, Senthilkumar Preethy, Samuel J.K. Abraham

**Affiliations:** a Dept of Paediatric Neurology, Jesuit Antonyraj memorial Inter-disciplinary Centre for Advanced Recovery and Education (JAICARE), Madurai, India; b Department of Paediatric Neurology, Kenmax Medical Service Private Limited, Madurai, India; c Department of Paediatric Neurology, Sarvee Integra Private Limited, Chennai, India; d Mary-Yoshio Translational Hexagon (MYTH), Nichi-In Centre for Regenerative Medicine (NCRM), Chennai, India; e Department of Immunology, The Tamil Nadu Dr. M.G.R. Medical University, Chennai 600032, India; f Department of Anesthesia, Thunder Bay Regional Health Sciences Centre, Thunder Bay, Ontario, Canada; g Dept. of Medical Life Science, Kyushu University of Health and Welfare, Japan; h Institute of Immunology, Junsei Educational Institute, Nobeoka, Miyazaki, Japan; i Centre for Advancing Clinical Research (CACR), University of Yamanashi - School of Medicine, Chuo, Japan; j Fujio-Eiji Academic Terrain (FEAT), Nichi-In Centre for Regenerative Medicine (NCRM), Chennai, India; k Antony, Xavier Interdisciplinary Scholastics (AXIS), GN Corporation Co. Ltd., Kofu, Japan; l Levy-Jurgen Transdisciplinary Exploratory (LJTE), Global Niche Corp., Wilmington, DE, USA

**Keywords:** Duchenne muscular dystrophy, N-163, Beta glucan, Disease modifying agent, Inflammation, Fibrosis, Dystrophin

## Abstract

**Background:**

This exploratory case-control study is to evaluate the effects of supplementation of *Aureobasidium pullulans*-N-163 strain produced 1,3–1,− 6 beta glucan in young patients with Duchenne muscular dystrophy (DMD).

**Methods:**

Twenty-seven male subjects aged 5–19 years with DMD were included, nine in the control arm and 18 in the treatment arm to receive N-163 beta glucan along with conventional therapies for 45 days. While performing the analysis, steroid usage was also taken into consideration, those not administered steroids (Steroid -ve) (Control, n = 5; treatment, n = 9), those administered steroids (Steroid +ve) (Control, n = 4; treatment, n = 9).

**Results:**

IL-6 showed a significant decrease in the treatment groups, especially the N-163 Steroid -ve group. IL-13 decreased in both treatment groups and TGF-β levels showed a significant decrease in the treatment groups, especially the N-163 Steroid –ve group, (p < 0.05). Dystrophin levels increased by up to 32% in the treatment groups compared to the control. Medical research council (MRC) grading showed slight improvement in muscle strength improvement in 12 out of 18 patients (67%) in the treatment group and four out of nine (44%) subjects in the control group.

**Conclusion:**

Supplementation with the N-163 beta glucan food supplement produced beneficial effects: a significant decrease in inflammation and fibrosis markers, increase in serum dystrophin and slight improvement in muscle strength in DMD subjects over 45 days, thus making this a potential adjunct treatment for DMD after validation.

**Trial registration:**

The study was registered in Clinical trials registry of India, CTRI/2021/05/033346. Registered on 5th May, 2021.

## Highlights

• Clinical study to evaluate the safety and efficacy of N-163 beta glucan in patients with Duchenne muscular dystrophy (DMD).
• After N-163 supplementation, there was decrease in inflammatory and fibrosis markers IL-6, IL-13 and TGF-β levels.
• Dystrophin levels in plasma increased by up to 32% in the N-163 group.
• Medical research council (MRC) grading showed muscle strength improvement in 67% of the patients.
• N-163 beta 1,3-1,6 glucan food supplement is a potential disease-modifying adjunct treatment for DMD.

## Introduction

Duchenne muscular dystrophy (DMD) is a devastating X-linked neuromuscular disorder causing severe and progressive weakness of skeletal muscles, leading to loss of ambulation along with concomitant impairment of cardiac and respiratory muscles and early mortality. Mutations in the dystrophin gene, which cause total loss of the dystrophin protein (Nagy et al., 2019), remain the major underlying mechanism. Loss of dystrophin leads to damage of the myofibers’ plasma membranes and distorts the structural stability of the plasma, leading to weakness in the myofibers. The weakened myofibers cannot withstand the contraction and relaxation cycles occurring during muscle function. The damage to the membrane releases the cytoplasmic contents, triggering the immune system and causing further muscle fibre damage, weakness and ultimately death (Rosenberg et al., 2015). A chronic proinflammatory state ensues, with neutrophil infiltration and macrophages’ phagocytosis of the degenerated tissue (Rosenberg et al., 2015), preventing repair of the muscle damage, which otherwise occurs in a highly orchestrated manner for faster repair in other physiological conditions. The muscle is relatively immunologically privileged, with a low capacity to generate localized immune responses and thus having low rates of abscess and granuloma formation (Rosenberg et al., 2015). Therefore, it becomes essential to modulate the inflammation and immunity to resolve the chronic inflammatory state in therapeutic approaches to DMD. Steroid therapy is the most employed immune-modulatory treatment approach. However, side effects include weight gain, weak bones, high blood pressure and behaviour changes in addition to muscle weakness and atrophy in the long term (Manzur et al., 2004, National Institute, 2021). Thus, there arises the need to develop strategies that will assist in immunomodulation with lesser side effects. Nutritional supplements are a potential option. Beside beta glucans yielding locomotor improvement in zebrafish models of DMD (Licitra et al., 2021), a 1–3,1–6 beta glucan from the N-163 strain of the black yeast *Aureobasidium pullulans* has been reported to decrease inflammation, evident by decreases in anti-inflammatory markers such as CD11b, serum ferritin, galectin-3 and fibrinogen. It also produces beneficial immune-modulation via a decrease in the neutrophil-to-lymphocyte ratio (NLR) and an increase in the lymphocyte-to-CRP ratio (LCR) and leukocyte-to-CRP ratio (LeCR) in human healthy volunteers (Ikewaki et al., 2021). Mitigation of lipotoxicity-associated inflammatory cascades in a mouse study has also been reported (Ikewaki et al., 2022). Another study done in an animal model of non-alcoholic steatohepatitis (NASH) showed a decrease in liver inflammation and accumulation of F4/80 + cells (macrophages associated with inflammation) (Ikewaki et al., 2022 Nov-Dec) in the liver. Therefore, we wanted to study the anti-inflammatory effects of this beta-glucan in DMD, as the prolonged activation of the innate immune response resulting in chronic inflammation, has been reported to be the factor underlying tissue damage in DMD (Rosenberg et al., 2015). The present exploratory case-control study is to evaluate the immunomodulatory, anti-inflammatory efficacy of the N-163 strain of *A. pullulans*-produced beta 1–3,1-,6 glucan in comparison with a conventional therapeutic regimen in patients with DMD.

## Methods

This trial was an investigator-initiated, single-centre, open-label, prospective, case-control clinical study of patients with DMD. The study was conducted over 45 days.

### Control arm

Control arm consisted of the existing standard of care treatment regimen comprised of standard routine physiotherapy for joint mobility along with medications, viz., T. calcium and vit. D 1000 with or without deflazacort (steroid) 6–24 mg.

### Treatment arm

One sachet of N-163 beta glucan (8 g gel, containing 48 mg of active ingredient) once daily along with conventional treatment.

### Inclusion criteria

Male subjects with molecular diagnosis of DMD aged 6–18 years who were willing to participate in the study with written informed consent.

### Exclusion criteria

Patients with a previous (within the past 1 month) or concomitant participation in any other therapeutic trial; a known or suspected malignancy; any other chronic disease or clinically relevant limitation of renal, liver or heart function according to the discretion of the investigator.

### Investigations

The following tests were carried out after written consent was obtained from the study subjects.

### At baseline and at the end of the study (after 45 days)

• Background survey: gender, date of birth, age, habits, current medical history, medication, treatment, allergies (to drugs and food), regular use of food for specified health uses, functional foods, health foods, intake of foods rich in β-glucan foods containing beta-glucan and intake of immunity-boosting foods
• Medical history and physical measurements: height, weight, BMI, temperature
• Physiological examination: systolic blood pressure, diastolic blood pressure, pulse rate
• ECG
• Muscle strength test using MRC grading (M.R.C., 2021)
• Six-minute walk test (6MWT) (McDonald et al., 2010)
• North Star Ambulatory Assessment (NSAA) (Scott et al., 2012)
• Blood sampling and investigations for the levels of IL-6, IL-13, TGF-β, creatinine kinase (CK), titin, haptoglobin, TNF-α, dystrophin, cystatin in the blood and myoglobin in the urine
• Subjects were contacted every week for drug compliance and recording of adverse effects, if any

***Study subjects =*** 28.

The study was designed as an exploratory case-control study, so there were two intervention conditions: one control and one treatment group. As the minimum number of participants required for statistical comparisons within and between intervention conditions is four per intervention condition, a total of 28 target study participants (10 in control group and 18 in treatment group [N-163]) were used. One patient was disqualified due to misrepresentation of diagnosis. Though two subjects had diagnosis of BMD which falls out of the inclusion criteria, the investigator felt that the subjects would benefit from the study, he applied for a waiver from the sponsor and notified the Independent Ethics Committee (IEC) about enrolling these two subjects who did not meet the inclusion criteria.

### Selection of study subjects

Study investigators and other investigators included study subjects who had consented to participate in the study, met the selection criteria and not the exclusion criteria, and who were judged to have no problem participating in the study.

### Primary outcome

Observation of changes in the levels of IL-6 in serum and myoglobin levels in urine from the baseline measured by ELISA.

### Secondary outcome

• Observation of changes in the levels of IL-13, TGF-β, CK, titin, dystrophin, haptoglobin and cystatin C in the serum measured by ELISA.
• Observation of changes in the muscle function tests
• Monitoring for adverse effects

#### ELISA

The biomarkers were validated using sandwich enzyme immunoassay. ELISA kits were purchased from different manufacturers and the assay details along with sensitivity are listed in Table 1. ELISAs were performed according to manufacturer’s instructions and the serum was diluted to fall within the linear range of each respective assay.

**Table 1:** Details of the ELISA kits along with their sensitivity.

**S.No**	**Human Antibody**	**Purchased from**	**Catalogue No.**	**Limit of detection/Sensitivity**
1	Interleukin-6 (IL-6)	Seimens	10995080	3.0 pg/ml
2	Myoglobin	Diagnostic Automation Inc	1667–18	270 pg/ml
3	Dystrophin (DMD)	KINESISDx	K12–1446	0.042 ng/ml
4	Titin (TTN)	Mybiosource	MBS762344	0.094 ng/ml
5	Creatinine Kinase (CK)	Agappe	51404001	2.0 U/L
6	IL-13	Diaclone	850080048	1.5 pg/ml
7	TGF-β	Diaclone	650010096	8.6 pg/ml
8	Cystatin C	Siemens	E0001V	0.06 µg/L
9	TNF-a	Siemens	LKNF1	6.23 pg/ml
10	Haptoglobin	Seimens	OSAV09	0.26 ng/ml

### Steroid Usage and analysis

During analysis of the data, we also studied the influence of steroids on the treatment outcome.

It has been reported to be a concern to include individuals who are taking corticosteroids in both the treatment and placebo groups because corticosteroids' beneficial effects may obscure the effects of the experimental medication (Merlini and Sabatelli, 2015). On the other hand, since corticosteroid medication is now the only known effective treatment for DMD patients (Merlini and Sabatelli, 2015), avoiding its use may be challenging or even unethical. Therefore we stratified subjects based on steroid use during the analysis of data as follows, (Steroid -ve) (Control, n = 5; treatment, n = 9), those administered steroids (Steroid +ve) (Control, n = 4; treatment, n = 9).

### Statistical analysis

All data were analysed using SPSS and Origin 2021b software. Results were expressed as means ± SD. ANOVA with Post-hoc Tukey’s HSD (honestly significant difference) were performed for statistical analysis as indicated. P values of < 0.05 were considered to be significant. Multiple regression analysis was also performed.

## Results

Twenty-eight patients were screened and 27 were assigned to control (n = 9) and treatment (n = 18). One patient was disqualified due to misrepresentation of diagnosis.

Demographics are shown in Table 2.

**Table 2:** Demographics and baseline characteristics.

Control Steroid -ve	Subject	Age in years	Weight in kgs	Exon Deletion	Clinical Diagnosis	Ambulatory/ Non-Ambulatory	Dose of Deflazacort (mg/day)	Duration of consumption of steroids
	2	6–10	27	46–55	Duchenne Muscular Dystrophy (DMD)	Non-Ambulatory	NA	NA
	3	16–20	48		Duchenne Muscular Dystrophy (DMD)	Ambulatory	NA	NA
	4	11–15	60	8–48	Duchenne Muscular Dystrophy (DMD)	Non-Ambulatory	NA	NA
	5	11–15	55	10–11/Dup	Duchenne Muscular Dystrophy (DMD)	Non-Ambulatory	NA	NA
								
Control Steroid +ve	1	16–20	65	44	Duchenne Muscular Dystrophy (DMD)	Non-Ambulatory	6	8 years
	2	6–10	25	5 & 6	Duchenne Muscular Dystrophy (DMD)	Ambulatory	12	2.5 months
	3	6–10	21	49–52	Duchenne Muscular Dystrophy (DMD)	Ambulatory	9	11 months
	4	11–15	48	52	Duchenne Muscular Dystrophy (DMD)	Ambulatory	6	6 years
								
N-163 Steroid -ve	1	6–10	28	48–50	Duchenne Muscular Dystrophy (DMD)	Ambulatory	NA	NA
	2	1–5	10	45–50	Duchenne Muscular Dystrophy (DMD)	Ambulatory	NA	NA
	3	11–15	38	45–50	Duchenne Muscular Dystrophy (DMD)	Ambulatory	NA	NA
	4	6–10	45	60	Becker muscular dystrophy (BMD)	Ambulatory	NA	NA
	5	6–10	39	10–11/ Dup	Duchenne Muscular Dystrophy (DMD)	Non-Ambulatory	NA	NA
	6	6–10	21	49–52	Duchenne Muscular Dystrophy (DMD)	Ambulatory	NA	NA
	7	11–15	39	43	Duchenne Muscular Dystrophy (DMD)	Non-Ambulatory	NA	NA
	8	11–15	40		Duchenne Muscular Dystrophy (DMD)	Non-Ambulatory	NA	NA
	9	6–10	22	44–57/Dup	Duchenne Muscular Dystrophy (DMD)	Ambulatory	NA	NA
N-163 Steroid +ve	1	1–5	18	48–50	Duchenne Muscular Dystrophy (DMD)	Non-Ambulatory	6	5 months
	2	11–15	59	17	Duchenne Muscular Dystrophy (DMD)	Ambulatory	6	2 months
	3	11–15	40	60	Becker muscular dystrophy (BMD)	Ambulatory	6	6 years
	4	6–10	20	48–52	Duchenne Muscular Dystrophy (DMD)	Ambulatory	6	3 years
	5	6–10	36	48–50	Duchenne Muscular Dystrophy (DMD)	Ambulatory	6	3 years

The stratification of patients was as follows based on steroid administration during analysis:

Group I: Control group (n = 9);.

A. Steroids not administered (n = 5) (Steroid -ve)
B. Steroids administered (n = 4) (Steroid +ve)

Group II: Treatment (N-163) group (n = 18);.

A. Steroids not administered (n = 9) (Steroid -ve)

A. Steroids administered (n = 9) (Steroid +ve)

The mean ± SD age for the total study population was 11.18 ± 3.86 years (range 5–19 years). The mean age across the groups were Control (Steroid-ve) − 14.25 years; Control (Steroid +ve) – 13.6 years; N-163 (Steroid -ve) – 9.6 years; N-163 (Steroid +ve) – 11.87 years. The mean ± SD body weight was 35.59 ± 15.5 kg (range = 10–65 kg).

### No adverse events were reported

**Fig. 1:**
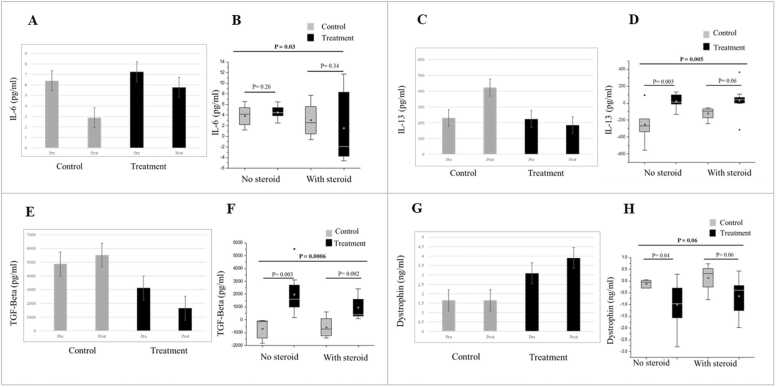
Post intervention-values of A. IL-6 showing decrease in treatment (N-163) and control groups; B. Mean difference of IL-6 levels from the baseline in control and treatment groups stratified based on steroid usage; No steroid, Control n = 5; treatment n = 9; With steroid, Control n = 4; treatment n = 9; C. IL-13 and E. TGF-Beta showing decrease in treatment (N-163 groups) but increase in control groups; D and F. Mean difference of IL-13 and TGF-Beta levels from the baseline in control and treatment groups stratified based on steroid usage; No steroid, Control n = 5; treatment n = 9; With steroid, Control n = 4; treatment n = 9; G: Post intervention-values of plasma dystrophin showing increase only in treatment (N-163) group; H. Mean difference of plasma dystrophin levels from the baseline in control and treatment groups stratified based on steroid usage. (*p-value significance < 0.05).

Regarding the primary outcome, IL-6 showed decrease in both the control group and in the treatment group (Fig. 1A, B). However, when the analysis was stratified for steroid administration, IL-6 showed the highest decrease in the N-163 Steroid -ve group, from a baseline value of 7.2 ± 1.2 pg/ml to 2.7 ± 0.03 pg/ ml post intervention compared to the control groups but a marginal increase was observed in treatment steroid+ve group from a baseline value of 7.29 ± 1.04 pg/ ml to 8.84 ± 6.39 pg/ ml (Fig. 1A, B).

IL-13 increased in both control groups—from 300.4 ± 114.5 pg/ml at baseline to 550.732 ± 107.95 pg/ml post-intervention in the Steroid -ve group and from 142 ± 112.82 pg/ml at baseline to 263.5 ± 99.38 pg/ml post-intervention in the Steroid +ve group. It decreased in both the treatment groups— Steroid -ve group, from 157.76 ± 148.68 pg/ml at baseline to 114.08 ± 81.5 pg/ml post-intervention and from Steroid +ve group 289.56 ± 232.88 pg/ml at baseline to 255.56 ± 214.13 pg/ml post-intervention (Fig. 1C,D). The decrease in the steroid-ve treatment group was significant when compared with the other groups (p-value=0.005).

TGF-β levels showed a significant decrease in the N-163 Steroid -ve group, from a baseline value of 3302 ± 1895 pg/ml to 1325.66 ± 517 pg/ml post intervention (Fig. 1E, F). This decrease was statistically significant (p-value=0.0006). TGF-Beta increased in the control groups post-intervention.

Dystrophin levels showed a significant increase in the N-163 Steroid -ve group, from a baseline value of 3.01 ± 1.58 ng/ml to 4.01 ± 1.44 ng/ml post intervention, and the N-163 Steroid +ve group went from a baseline value of 3.15 ± 2.43 ng/ml to 3.78 ± 2.17 ng/ml post intervention. The N-163 Steroid -ve group showed higher dystrophin expression than the N-163 Steroid +ve group with the difference being significant (p-value =0.06) (Fig. 1G,H). The percentage increase in dystrophin levels in the treatment group was up to 32.8%. The difference between the control and treatment groups was significant both in steroid-ve groups (p-value =0.04) and steroid +ve groups (p-value =0.06).

**Fig. 2:**
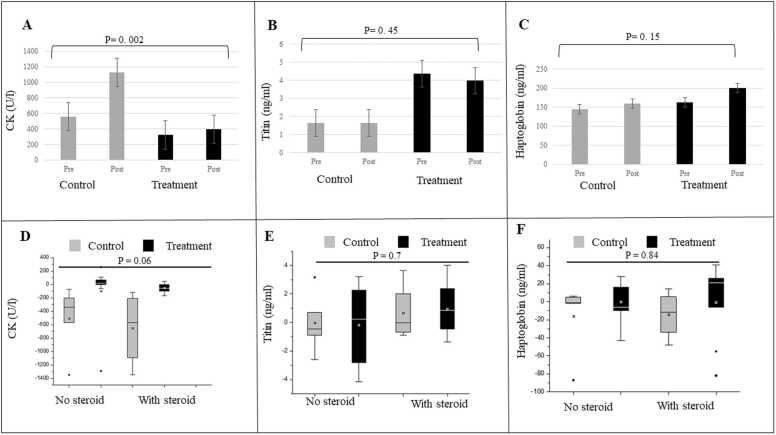
A-C: Levels of Creatinine kinase (CK), Titin and Haptoglobin between control and treatment group (N-163), pre- and post-intervention; D-F: Mean difference of CK, Titin and Haptoglobin levels from the baseline in control and treatment groups stratified based on steroid usage; No steroid, Control n = 5; treatment n = 9; With steroid, Control n = 4; treatment n = 9. (*p-value significance < 0.05).

**Fig. 3:**
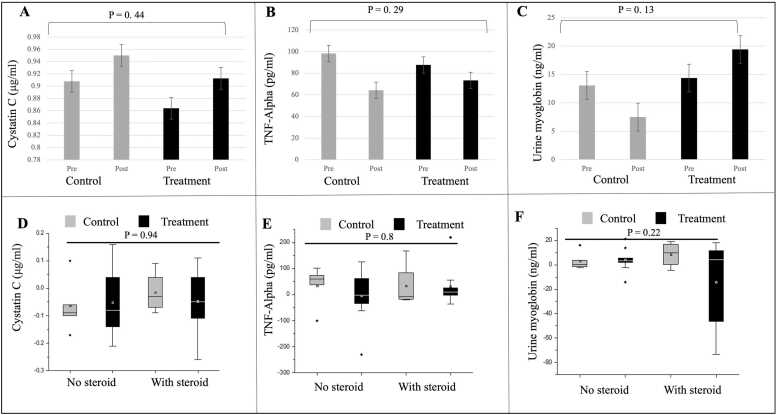
A-C: Levels of Cystatin C, TNF-Alpha and urine myoglobin between control and treatment group (N-163), pre- and post-intervention (*p-value significance < 0.05); D-F: Mean difference of Cystatin C, TNF-Alpha and urine myoglobin levels from the baseline in control and treatment groups stratified based on steroid usage; No steroid, Control n = 5; treatment n = 9; With steroid, Control n = 4; treatment n = 9. (*p-value significance < 0.05).

CK increased in the control (Steroid -ve: from baseline level of 666.60 ± 324.42 U/l to 1173.64 ± 492.07 U/l, post-intervention, Steroid +ve: from 419.25 ± 317.56–1070.33 ± 785.29 U/l) and treatment groups (Steroid -ve: from 376.44 ± 235.77 U/l to 473.78 ± 648.29 U/l; Steroid +ve: from 266.11 ± 124.85 U/l to 319.70 ± 116.62 U/l (Fig. 2A,D). Titin decreased in both the treatment (N-163) groups (steroid-ve: from baseline value of 4.91 ± 2.65 ng/ml to post-intervention 5.10 ± 3.13 ng/ml; steroid +ve: from 3.82 ± 3.22 ng/ml to 2.85 ± 2.96 ng/ml) and in the control groups (steroid-ve: from 5.16 ± 4.39 ng/ml to 5.18 ± 3.17 ng/ml; steroid+ve: from 6.45 ± 3.39 ng/ml to 5.79 ± 3.41 ng/ml) but the difference among the four groups post-intervention was not significant (Fig. 2B,E). Haptoglobin (Fig. 2C, F) and Cystatin C (Fig. 3A,D) increased in the treatment and control groups, Haptoglobin (treatment: steroid-ve: from 109.22 ± 15.09 ng/ml to 109.22 ± 30.28 ng/ml; steroid+ve: from 152.89 ± 30.30 ng/ml to 153.56 ± 41.18 ng/ml; control: steroid-ve: from 137.40 ± 63.65 ng/ml to 153.20 ± 55.43 ng/ml; steroid+ve: from 153.25 ± 53.63 ng/ml to 167.50 ± 46.98 ng/ml); Cystatin C (treatment: steroid-ve: from 0.88 ± 0.12 μg/ml to 0.93 ± 0.05 μg/ml; steroid +ve: from 0.85 ± 0.11 μg/ml to 0.89 ± 0.10 μg/ml; control: steroid-ve: from 0.90 ± 0.21 μg/ml to 0.96 ± 0.16 μg/ml; steroid+ve: from 0.92 ± 0.06 μg/ml to 0.93 ± 0.08 μg/ml) but the difference among the four groups post-intervention was not significant. TNF-α decreased in treatment (steroid+ve: from 88.71 ± 83.91 pg/ml to 56.82 ± 38.94 pg/ml and control groups (steroid-ve: from 85.30 ± 49.82 pg/ml to 50.87 ± 38.32 pg/ml; steroid+ve: from 114.36 ± 124.74 pg/ml to 81.05 ± 53.89 pg/ml) but increased in treatment steroid-ve group ( from 86.53 ± 75.37 pg/ml to 89.88 ± 81.29 pg/ml) (Fig. 3B,E). Urine myoglobin decreased in the treatment (steroid-ve: from 14.62 ± 11.73 ng/ml to 10.65 ± 4.50 ng/ml) and control group (steroid-ve: from 10.82 ± 12.66 ng/ml to 7.61 ± 7.09 ng/ml; steroid+ve: from 15.86 ± 10.71 ng/ml to 7.39 ± 2.43 ng/ml) but increased in the treatment (steroid+ve: from 14.12 ± 7.48 ng/ml to 28.16 ± 28.39 ng/ml) group. However, the difference was not significant. (Fig. 3C,F).

**Fig. 4:**
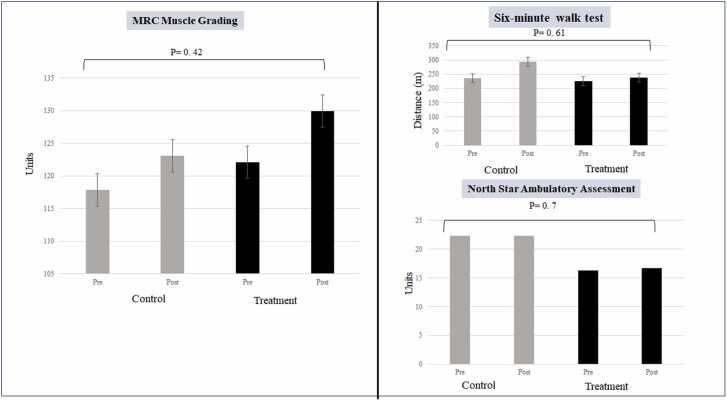
MRC muscle grading, 6MWT and NSAA results between control and treatment group (N-163) post-intervention (*p-value significance < 0.05).

The MRC grading showed slight improvement in 12 out of 18 patients (67%) in the treatment group and only in four out of nine (44%) subjects in the control group (Table 3, Fig. 4). The comparison between groups regarding 6MWT and NSAA showed insignificant relationship between the groups (Fig. 4).

**Table 3:** Medical research council (MRC) grading of muscle power in the study groups.

**Subject**	**Ambulatory/Non-ambulatory**	**Baseline**	**Post-intervention**	**Progression**
**Control group**				
1	Ambulatory	151	165	Improved
2	Ambulatory	133	162	Improved
3	Non-ambulatory	70	80	Improved
4	Non-ambulatory	164	164	No change
5	Non-ambulatory	109	106	Worsened
6	Non-ambulatory	102	105	Improved
7	Non-ambulatory	117	115	Worsened
8	Non-ambulatory	110	109	No change
9	Non-ambulatory	105	102	Worsened
**Treatment Group**				
1	Ambulatory	134	133	Worsened
2	Ambulatory	146	163	Improved
3	Ambulatory	119	131	Improved
4	Ambulatory	127	136	Improved
5	Ambulatory	154	160	Improved
6	Ambulatory	158	168	Improved
7	Non-ambulatory	102	119	Improved
8	Non-ambulatory	116	117	Improved
9	Non-ambulatory	127	127	No change
10	Non-ambulatory	120	131	Improved
11	Non-ambulatory	96	107	Improved
12	Non-ambulatory	111	122	Improved
13	Non-ambulatory	117	113	Worsened
14	Non-ambulatory	108	108	No change
15	Non-ambulatory	103	107	Improved
16	Non-ambulatory	145	148	Improved
17	Non-ambulatory	93	119	Improved
18	Non-ambulatory	Test couldn’t be performed

Regression analysis showed that there was no significant correlation of the steroid usage between the two control groups and between the N-163 treatment groups on the values of IL-6, IL-13, TGF-β, Dystrophin, 6MWT and NSAA (Table 4).

**Table 4:** Regression analysis of the biomarkers and muscle function test results post-intervention in the four groups (N-163 Steroid-ve, N-163 Steroid+ve, Control Steroid -ve and Control Steroid +ve).

**Coefficients**^**a**^						
Model		Unstandardized Coefficients		Standardized Coefficients	t	Sig.
		B	Std. Error	Beta		
1	Constant – Post values	3.803	2.528		1.504	0.271
	IL-6	-0.200	0.323	-0.241	-0.618	0.599
	IL-13	-0.001	0.003	-0.115	-0.186	0.870
	TGF β	0.000	0.000	0.862	1.136	0.374
	Dystrophin	-0.347	0.216	-0.594	-1.606	0.249
	6MWT	0.012	0.016	0.694	0.744	0.534
	NSAA	-0.185	0.250	-1.014	-0.742	0.536

Dependent Variable: Groups (N-163 Steroid-ve, N-163 Steroid+ve, Control Steroid -ve and Control Steroid +ve

## Discussion

Current interventions for DMD, such as corticosteroids and rehabilitative care, help to prolong survival up to the third or the fourth decade of life. Corticosteroids remain the mainstream supportive approach to slow inflammation and the associated decline in muscle strength and function (Manzur et al., 2004). However, steroids have their own adverse effects, and their prescription is based on risk versus benefit to that specific patient and tolerance to the medication. Exon-skipping gene therapy and cell-based strategies to replace the mutant DMD gene are in development, but the desired outcome has not yet been achieved. In the meantime, nutraceuticals can be considered potential strategies for immune modulation and alleviating inflammation, as they are safer with lesser adverse effects (Manzur et al., 2004). Improvement of the locomotor performances and mitochondrial respiration by 1,3–1,6 beta-glucans in zebra fish model of muscular dystrophy (Licitra et al., 2021) has already been reported.

In the current study, we focussed on a 1–3,1–6 beta glucan from the N-163 strain of the black yeast *A. pullulans* that has been reported to mitigate inflammation, evidenced by a decrease in anti-inflammatory markers and production of beneficial immuno-modulation (Licitra et al., 2021, Ikewaki et al., 2021, Ikewaki et al., 2022). The safety profile of N-163 beta glucan has been confirmed by the results.

### Anti-inflammatory and anti-fibrotic outcomes

Circulating IL-6 is chronically elevated in individuals with DMD (Stephenson et al., 2020), which has been reported to contribute to DMD-associated cognitive dysfunction. IL-6 blockades have been advocated as a therapeutic approach for DMD (Mammen and Sartorelli, 2015). In the present study, IL-6 showed highest decrease in the N-163 Steroid -ve group (Fig. 1B). While IL-6 is an acute inflammatory biomarker (Mann et al., 2011), IL-13 is a pro-fibrotic biomarker (Mann et al., 2011) and was significantly decreased (Fig. 1C,D). Together with the TGF-β pathway, it is a major proinflammatory and pro-fibrotic cytokine responsible for the chronic inflammatory response leading to replacement of the muscle by scar tissue or fibrosis, resulting in muscle weakness and loss of muscle function (Ceco and McNally, 2013). IL-6 decrease in control groups matched that of DMD patients who were treated with corticosteroids in earlier studies (Pelosi et al., 2015). The increase in IL-6 in the steroid+ve N-163 group, we hypothesize could be attributed to beneficial satellite stem cell mediated muscle regeneration akin to what happens after exercise in healthy young adults (Snijders et al., 2015). However, this needs further evaluation to understand the dynamics. TGF-β levels also showed a significant decrease in the N-163 Steroid -ve group (Fig. 1E,F). Dystrophin restoration of 20% expression (Pelosi et al., 2015, Shimizu-Motohashi et al., 2019) is considered the point of efficacy for a DMD therapy (Mendell et al., 2012) and was found to increase by 32.8% in both the treatment groups (Fig. 1G,H) of the present study from baseline. We used serum dystrophin evaluation as recent evidence points to the fact that the lack of dystrophin in the smooth muscle of blood vessels is the major contributing factor to skeletal muscle pathogenesis in DMD and not the dystrophin deficiency in skeletal or cardiac muscle (Wells, 2019). Dystrophin is expressed in the tunica media of blood vessels, especially in the muscular arteries. It has also been reported that the blood vessel-associated dystrophin is encoded by another gene than the skeletal muscle dystrophin gene (Gajendran, 2018). In a mdx mice model study, it was shown that dystrophin which was absent in the blood vessels could be restored by induced genetic expression of full-length human dystrophin (h-dys) and this expression was able to restore the NO-dependent attenuation of α-adrenergic vasoconstriction in the exercising muscles, preventing further exacerbation of muscle fibre necrosis (Augier et al., 1992 Feb). In the present study, an increase in the plasma levels of dystrophin, which must have been derived from vascular smooth muscle is evidence for the efficacy of beta glucans as a potential adjunct to restore dystrophin in DMD. However, this hypothesis needs validation in future research.

### Other biochemical markers of relevance

While haptoglobin and urine myoglobin did not show significant differences, the increase in urine myoglobin in the N-163 Steroid +ve group deserves more analysis concerning the underlying mechanism. Greater activity among steroid-treated individuals may place their dystrophin-deficient muscles under greater mechanical stress, predisposing them to further muscle fibre damage and consequent myoglobinuria (Ito et al., 2006). While titin decreased in the N-163 steroid +ve group and in the control Steroid +ve group, there was an increase in CK, which is paradoxical, as reports suggest that titin concentration correlates significantly with serum CK concentration (Awano et al., 2018).

### Muscle strength evaluation

There were three evaluations to assess muscle strength and tone, done in a blinded manner by the same physiotherapist at baseline and post intervention. Though the 6MWT and NSAA did not show any significant differences between the groups, MRC grading showed improvement of muscle strength in 67% of the subjects in the treatment group compared to 44% subjects in the control group, which is significant. The limitation of this being a 45-day study is relevant to the muscle-strength and functional evaluations, mandating the need for a longer study and follow-up duration. However, though small, the improvement in MRC grading at 45 days could be again attributed to the immune modulation effects of this disease-modifying supplement. The study shows proof of concept that DMD could be tackled by the N-163 beta glucan from three aspects: decrease in inflammation shown by decreased IL-6 and TNF-α, decrease in fibrosis evident by decreased TGF-β and IL-13 and, more importantly, restoration of dystrophin evident from a 32.8% increase in dystrophin levels. These effects hold regardless of the use or non-use of steroids (Table 4), which is important, as this safety-proven food supplement can help DMD patients regardless of steroid status.

Chronic inflammation being common to pathogenesis of all muscular dystrophies, immunomodulatory treatment may benefit patients with diverse types of muscular dystrophy (Garrood et al., 2008). Further, modulating the inflammatory response and inducing immunological tolerance to de novo dystrophin expression is critical to the success of dystrophin-replacement therapies (Raimondo and Mooney, 2021). The need to evaluate the muscles involved in respiratory function and myocardium should be mentioned here, as they are the cause of mortality in most of the DMD patients (Theadom et al., 2014). Though other dystrophies, such as limb girdle muscular dystrophy (LGMD), do not involve respiratory or cardiac muscles, inflammatory overactivity is the common pathophysiology among types of muscular dystrophy (Garrood et al., 2008). Once proven efficacious for DMD, extending the beneficial application of the N-163 beta glucans to other dystrophies such as LGMD can be considered.

DMD is a rare genetic disease with a maximum life expectancy of up to fourth decade, with the majority of victims dying in their late twenties to thirties. The average lifespan at birth, which was 20 + years for those born in or before 1970, has gradually increased by 10–15 years for those born and diagnosed with DMD in the 1980 s and 1990 s. This increase is attributed to better or early ventilatory assistance, steroid usage and cardiac care (Thomas et al., 2012, Ryder et al., 2017), which are only supportive interventions. With the gene therapies approved recently, there is a hope of additional progress and increase in lifespan (Kieny et al., 2013). Though these gene therapies (such as exon skipping) address the root cause by splicing out selected exons from the pre-mRNA at or next to the mutation site, generating a translatable transcript from the mutant dystrophin gene leading to dystrophin expression (Duan, 2018, Echevarría et al., 2018), they are still marred by challenges such as delivery of gene-editing components throughout the musculature and mitigation of possible immune responses (Dzierlega and Yokota, 2020, Olson, 2021). The current need, therefore, is to modulate the immune system and control the inflammation and ensuing fibrosis to delay the progression of the disease. The earlier usage of steroids in a regular manner was later changed to intermittent usage (Kourakis et al., 2021) with regimens varying between institutes; now, newer steroids with lesser adverse effects are in various stages of progress towards clinical applications (Boziki et al., 2020). In this background, the safety of this N-163-produced beta glucan food supplement without adverse reactions is to be considered an indispensable value addition. Targeting the inflammation component (the criteria for selecting this supplement for this study) having yielded beneficial outcomes, additional studies on this characteristic could be of value to possibly extending their application for other neuroinflammatory diseases, such as multiple sclerosis.

The possible mechanisms by which the N-163 strain of *A.pullulans* produced beta-glucan produced the anti-inflammatory, anti-fibrotic beneficial effects may be attributed to its effects on Peroxisome proliferator-activated receptor (PPAR)- γ (Ikewaki et al., 2022 Nov-Dec). N-163 strain produced beta-glucan has been shown to regulate inflammatory markers and immunity, namely IL-6, C-reactive protein (CRP), D-Dimer, ferritin, neutrophil-to-lymphocyte ratio (NLR), lymphocyte-to-C-reactive protein ratio (LCR), leucocyte-to-C-reactive protein ratio (LeCR), and leukocyte-to-IL-6 ratio (LeIR) in previous pre-clinical and clinical studies of diabetes, Non-alcoholic steatohepatosis (NASH) and COVID-19 (Ikewaki et al., 2021, Ikewaki et al., 2022, Ikewaki et al., 2022 Nov-Dec, Raghavan et al., 2022a). The N-163 strain produced beta-glucan has been earlier shown to decrease inflammation, produce beneficial immunomodulation via the gut-brain axis as well (Preethy et al., 2022, Raghavan et al., 2022b).

The *A.pullulans* produced beta-glucan has been in human consumption for more than two decades and its safety has been established in healthy volunteers apart from in conditions such as autism spectrum disorder (ASD), NASH, COVID-19, diabetes and dyslipidaemia (Ikewaki et al., 2021, Ikewaki et al., 2022, Raghavan et al., 2022a, Preethy et al., 2022, Raghavan et al., 2022b, Ganesh et al., 2014, Dedeepiya et al., 2012).

The limitations of the study include uneven distribution of subjects, broad age range (5–19 years) and short follow-up (only 45 days); improvements in muscle function over the course of the study showed variability that may have been due to the level of sensitivity to change of functional assessments during the disease progression in the age group. Among the 27 subjects, two-thirds were ambulatory and the remaining non-ambulatory; the evaluation criteria differences must be kept in mind, which may show equivalent quantification among all DMD patients at different stages of disease severity when non-invasive myograms to measure the individual muscles accurately could be undertaken. Further, the steroid dose was not uniform and fell into a broad range (6–24 mg). Also, the consumption of steroids vs those who did not consume them or those who had stopped steroids after an initial duration of consumption, as well as regimen variation, are to be considered while interpreting the outcomes. All these aspects mandate the need for larger randomized clinical trials of longer duration to validate this supplement as a treatment.

## Conclusion

N-163 beta glucan with and without steroids helped decrease IL-6, TGF-β and IL-13 and increase dystrophin levels along with improvement of muscle strength in subjects with DMD in this clinical study. While the benefits documented may help slow the rate of progression of this devastating disease, confirmation by longer and larger studies will help establish this agent as a disease-modifying agent for DMD. After such validation, extending its application to other dystrophies such as LGMD could be considered, and further in-depth research in neuroinflammatory diseases are likely to shed light on the mechanism of action, leading to additional beneficial applications.

## Ethics approval and consent to participate

The study was registered in Clinical trials registry of India, CTRI/2021/05/033346. Registered on 5th May, 2021. The study was approved by the Institutional Ethics Committee (IEC) of Saravana Multispeciality Hospital, India on 12th April, 2021.

## Funding

No external funding was received for the study.

### Consent for publication

Not applicable.

## CRediT authorship contribution statement

**Kadalraja Raghavan**: Conceptualization and Data curation. **Samuel JK Abraham**: Conceptualization, Methodology, Writing - Original Draft, Supervision and Project administration. **Nobunao Ikewaki**: Conceptualization. **Senthilkumar Preethy**: Writing - Original Draft. **Rajappa Senthilkumar**: Formal analysis. **Vidyasagar Devaprasad Dedeepiya**: Formal analysis. **Subramaniam Srinivasan**: Writing - Review & Editing. **Subramanian Pushkala**: Writing - Review & Editing. **Sudhakar S Bharatidasan**: Writing - Review & Editing. **Masaru Iwasaki**: Writing - Review & Editing.

## Data Availability

All data generated or analysed during this study are included in the article itself.
